# Characteristics of Acute Childhood Illness Apps for Parents: Environmental Scan

**DOI:** 10.2196/29441

**Published:** 2021-10-19

**Authors:** James Benoit, Lisa Hartling, Michelle Chan, Shannon Scott

**Affiliations:** 1 Faculty of Nursing University of Alberta Edmonton, AB Canada; 2 Department of Pediatrics Faculty of Medicine and Dentistry University of Alberta Edmonton, AB Canada

**Keywords:** internet, mHealth, mobile health, digital health, ehealth, app, mobile application, Android, Apple, marketplace, environmental scan, review, acute childhood illness, knowledge translation, child, parent, caregiver, mobile phone

## Abstract

**Background:**

Providing parents with resources that aid in the identification and management of acute childhood illnesses helps those parents feel better equipped to assess their children’s health and significantly changes parental health-seeking behaviors. Some of these resources are limited by accessibility and scalability. Remote locations and staffing limitations create challenges for parents aiming to access their child’s health information. Mobile health apps offer a scalable, accessible solution for improving health literacy by enabling access to health information through mobile devices.

**Objective:**

The aim of our study is to create an inventory of acute childhood illness apps that are available to North American parents and caregivers, assess their quality, and identify the areas in which future apps can be improved.

**Methods:**

We conducted an environmental scan to identify and summarize app information for parents and digital health researchers. The Google and Apple app marketplaces were used as search platforms. We built a list of search terms and searched the platforms for apps targeted at parents and related to acute pediatric illnesses in the United States and Canada. We assessed apps meeting the inclusion criteria using the Mobile App Rating Scale (MARS), a validated tool for assessing the quality of health apps. The MARS examines apps on 5 subscales: engagement, functionality, aesthetics, information quality, and subjective quality. Data were analyzed by MARS subscale averages and individual item scores.

**Results:**

Overall, 650 unique apps were screened, and 53 (8.2%) were included. On a scale of 1-5, apps had an average engagement score of 2.82/5 (SD 0.86), functionality score of 3.98/5 (SD 0.72), aesthetics score of 3.09/5 (SD 0.87), information quality score of 2.73/5 (SD 1.32), and subjective quality score of 2.20/5 (SD 0.79). On the same scale of 1-5, app scores ranged from 2.2/5 to 4.5/5 (mean 3.2, SD 0.6). The top 3 MARS-scored apps were *Baby and Child First Aid* (4.5/5), *Ada* (4.5/5), and *HANDi Paediatric* (4.2/5). Taken together, the top 3 apps covered topics of emergency pediatric first aid, identification of (and appropriate response to) common childhood illnesses, a means of checking symptoms, and a means of responding to emergency situations. There was a lack of Canadian-based app content available to parents in both marketplaces; this space was filled with content originating primarily in the United Kingdom and the United States. In addition, published evidence of the impact of the included apps was poor: of 53 apps, only 5 (9%) had an evidence base showing that the app had been trialed for usability or efficacy.

**Conclusions:**

There is a need for evidence-based acute childhood illness apps of Canadian origin. This environmental scan offers a comprehensive picture of the health app landscape by examining trends in acute childhood illness apps that are readily available to parents and by identifying gaps in app design.

## Introduction

### Background

The unexpected COVID-19 outbreak has affected how health information is communicated, how individuals seek health information and services, and how these services are delivered [1,2]. Families are delaying seeking health care for children, introducing consequences for their current and future health outcomes by delaying emergency care and wellness visits [3-6]. Notably, there has been a 57% reduction in pediatric emergency department (ED) use, with use inversely correlated with pandemic severity [7]. Although on the surface this may look encouraging, this decrease was primarily due to a drop in low-acuity visits [7]. Providing resources to parents that aid in the detection and identification of childhood illnesses helps them to feel better equipped to assess their child’s health and significantly changes parental health-seeking behaviors [8]. For context, these illnesses could include conditions such as gastroenteritis, bronchiolitis, and asthma.

Traditionally, parents obtained health information primarily from friends, family, and physicians [9,10]. In clinical settings, conventional modes of communicating complex health information to parents include information sheets and pamphlets [11]. In Canada, another common health resource for parents is teletriage, accessed by calling 811 in all but one province and one territory (Manitoba and Nunavut, respectively), where nurses answer health- and illness-related questions. This service is highly used: for example, in the province of Alberta, 694,313 calls were made among a population of 4.3 million in 2018 [12]. However, more recent studies in health information–seeking behavior suggest that this may be changing, with an Australian pediatric children’s hospital survey indicating that 96% of the parents use the internet and 63% use a smartphone to search for health information [13]. In conjunction with Google searches, this behavior is used to establish a cause of illness, access means of assessing symptom severity, and exercise prudence regarding visiting a physician [14]. Parents also search for support through web-based forums, and this means of information gathering provides additional reassurance and validation [15].

However, some of these information resources are limited by accessibility and scalability. Remote locations (ie, geography) and staffing limitations create challenges for parents aiming to access health information about their child [16,17]. These challenges are compounded by the existing variance in parents’ health literacy and language skills [11] and their willingness to ask others for health information; for example, many parents feel unable to express their anxiety about their child’s health because of fear that they will be perceived as worriers [18,19]. In addition, 30% of the Canadian parents who have children presenting to the ED have low health literacy [20], which accounted for 940,637 of the 3,135,457 children’s visits to Canadian EDs in 2018 [21]. Notably, parents with low health literacy are 3 times as likely to bring their child in for nonurgent conditions [22]. It is therefore vital to the mission of delivering equitable health care that resources are made accessible to parents with lower health literacy.

Mobile health (mHealth) apps offer a potential solution for improving health literacy by enabling access to health information through a new medium: the app marketplaces found on mobile devices such as phones and tablets [23]. A large study of 4974 American adults demonstrated that significant associations exist between adequate health literacy and the use of health information technology such as patient portal apps [24]. More than 96% of Americans [25] and 87% of Canadians [26] own a mobile phone, and smartphones are owned by 81% and 78%, respectively. Of mobile phone owners, more than 97% use an Android- or Apple-based operating system [27]. In 2017, nearly a third of the Canadian adults used mHealth apps to monitor their health [28]. Importantly, increased use of health apps has been shown to have a significant correlation with improved health behaviors [29].

Previous studies examining parents’ internet search patterns for health information have indicated that a variety of information-seeking strategies are used and that information is trusted differently based on its source (eg, information found from searching university- and hospital-based websites was considered far more accurate and safer than information found within public search engine results) [30]. However, little is known about parents’ interactions with health apps. A recent review suggested that existing app assessment tools are targeted at expert users, and nonexperts such as parents and caregivers still lack these resources to make an informed decision about which apps to use [31]. An investigation of patient-facing apps showed that the participants were unable to complete 57% of the relevant tasks because of a lack of confidence with the app and frustration with its design and navigation, but paradoxically they remained interested in using apps to self-manage care [32].

There are differing opinions on the current use of apps for parents. It has been pointedly suggested that “apps don’t help parents of sick kids” [33]. However, this viewpoint drew its conclusions from a review that included only 3 digital interventions found in scientific studies [34]. As another review of apps pointed out, there is little crossover between scientific studies and app marketplaces: in a review of pain-related apps, those seen in the marketplace are not seen in scientific studies and vice versa [35]. Given the large and increasing number of health-related apps available (more than 300,000 in 2017 [36,37]), there is a disparity between the size of the health app landscape and its representation in scientific studies. To create an actionable set of information for parents and caregivers, it will be useful to look beyond apps that appear in scientific studies.

### Health App Landscape

To create an inventory of the apps available to parents in North America that provide acute pediatric health information, we conducted an environmental scan, a review technique rooted in business that is designed to summarize information for decision-makers [38,39]. Environmental scans are conducted to identify trends and avoidable pitfalls in a specific area of inquiry, be it related to product, policy, or strategy. We used the results of this scan to create a picture of the health app landscape by examining trends in apps related to acute pediatric health that are readily available to parents and identifying the gaps in app design that can be addressed.

## Methods

### Overview

To carry out a structured environmental scan, we designed a search strategy that worked with the algorithms of Google’s and Apple’s app marketplaces. The finalized search strategy was applied by 2 reviewers (JB and MC) to these 2 app marketplaces in Canada and the United States. Next, 2 reviewers (JB and MC) screened the apps based on predefined inclusion criteria and extracted attributes about each app. We built evidence tables describing app characteristics (eg, number of reviews) and app quality (eg, quality of information) using items from the Mobile App Rating Scale (MARS) [40] and analyzed the results for trends. It should be noted that although this approach includes elements of previously suggested search strategies for apps (eg, the 7 strategies for assessing apps proposed in the study by Boudreaux et al [41]), some elements of such a strategy (eg, searching app clearinghouses) are no longer widely used, and others (eg, piloting the apps) are beyond the scope of this environmental scan.

### Search Strategy

We designed a set of 17 search terms in collaboration with a research librarian to identify relevant apps in the Google Play Store and Apple App Store. We limited our searches to these stores, given that the scope of this scan was to investigate apps that are likely to be accessible to North American parents. As other environmental scans that were drawn from for our approach have discussed [42], there is little formal knowledge publicly available about the specifics of how either store’s search function works, beyond testing for specific functionality (eg, the Google Play Store’s search used Boolean terms, whereas the Apple App Store’s did not). The results presented here will be *best available* knowledge based on internet searches and responses to information requests.

On the basis of responses to the queries sent to the Google Play Store and Apple App Store support teams, we learnt that the Google Play Store app search currently integrates data from title, publisher, and app descriptions. The results displayed are then limited by their search rank for the specific search term used. The Apple App Store app search has different input parameters: it uses app title, keywords, and primary category to search, whereas the app’s promotional text does not affect search rank and the search does not use the app’s full description. However, keywords are not displayed for apps, making it difficult to expand search terms directly based on visible information.

Apps not available in a particular country’s store or on a particular device were not visible in the results. Similarly, personalization of results (demonstrated by 2 users entering the same search term and generating different lists of results based on hidden user metrics) makes it difficult for a researcher to be sure that their queries are returning all relevant apps. We addressed this issue by building software using the Google Play Store’s and the Apple App Store’s application programming interfaces (the intermediary that communicates among different pieces of software) for both the Canadian and US stores and conducted tests to ensure that personalized results were not being returned and that the same set of results would be returned when different users ran the same search. This approach to remove result personalization addresses the inconsistency of personalized search results among users.

We examined the first 10 apps relevant to our scan that appeared using plain-language search terms (eg, child illness) for vocabulary in the stores’ description related to the app’s intended purpose and compiled a list of 17 search terms using language that appeared frequently: *Child illness*; *Pediatric illness*; *Paediatric illness*; *Child symptoms*; *Pediatric symptoms*; *Paediatric symptoms*; *Child care illness*; *Pediatric care illness*; *Paediatric care illness*; *Child diagnosis*; *Pediatric diagnosis*; *Paediatric diagnosis*; *Parent care child*; *Parent child illness*; *Child carer*; *Pediatric carer*; *Paediatric carer*.

### Search and Screening

These terms were searched 4 times on September 14, 2020, for 4 marketplace locations (the US Apple App Store, US Google Play Store, Canadian Apple App Store, and Canadian Google Play Store), and a maximum of 50 results per search term per store returned (for a maximum of 850 apps returned per store location across all searches). We built custom software in Python (Python Software Foundation) that used app marketplace application programming interfaces and stored search results in a comma-separated values database. We confirmed with test searches that the same set of results was returned on 2 different devices. The results were imported into an Excel spreadsheet (Microsoft Corporation). The MARS was adapted to a spreadsheet-ready format to allow for ease of charting.

Three inclusion criteria were used to screen apps:

1. The app contained content related to acute child (age 0-21 years) illness.
2. The app’s intended audience included parents or informal carers (eg, non–health care professionals).
3. The app still existed in the Google Play Store or Apple App Store when being assessed.

In addition, ad hoc reasons were recorded when apps included in the screening process could not be assessed with the MARS (eg, once downloaded, the app contained no content).

The title and description of each app were independently assessed by 2 researchers (JB and MC) for inclusion. In cases of ambiguity or where disagreement occurred after the results were compared, photos of the app depicting examples of its appearance and content on the Google Play Store or Apple App Store were used to gather additional information for inclusion and exclusion. The 2 researchers discussed this information to reach consensus.

### App Quality Assessment

We based the app assessments on the MARS, a validated tool designed for health app assessment [40,43]. The MARS has high internal consistency and interrater reliability, designed to classify and rate the quality of novel health apps; it is also used as a tool for guiding app design. The MARS assesses app quality based on 5 subscales: engagement, functionality, esthetics, information quality, and subjective quality. Each section includes a number of items that are directed questions. All items are scored out of 5, with a higher score indicating a better outcome. Each subscale score is determined by taking a mean of the subscale item scores. The MARS subscales can be found in its entirety in Multimedia Appendix 1.

To determine interrater agreement, 2 reviewers (JB and MC) conducted the MARS assessment for 10% of the apps, consistent with previous work [44]. We calculated the interrater agreement by identifying occurrences of major differences in the item scores (differences greater than 1) [42] and assessing the agreement between the 2 reviewers using the Cohen κ [45]. After excluding one app from comparison because of an unresolvable difference in app accessibility among the test devices, the interrater agreement among the remaining app items was found to be substantial at 0.69 [46], above the 0.60 threshold indicating that significant disagreements exist [47], and 1 reviewer conducted the remaining assessments.

Individual app assessments were conducted by installing each app on an Android or Apple device (if the same app was available on both devices, we compared the 2 versions for major differences and assessed the Android version). The apps were then launched and browsed for 10 minutes. When necessary, an account was created for apps that required a log in to access the app’s content. After exploring the app, the MARS was applied, with the app accessed as necessary to gather information for specific scale items. The characteristics of apps that the MARS collects include information such as the app’s rating, date of last update, and intended age group; a complete list is shown in Multimedia Appendix 1, Table S1. The 23 MARS items, grouped by subscale, are presented in Multimedia Appendix 1, Table S2. To determine whether the app appeared in the literature, Google Scholar was searched for occurrences of the app’s name.

### Analyses

The item scores for app quality were averaged across dimensions to create a set of 5 scores for each app. The app scores were averaged across items in the engagement, functionality, esthetics, and information subscales to create a final app score. Data were treated as interval level data, consistent with the recommendations by Norman around the increased robustness of parametric tests [48,49]. The scores were converted to a score out of 5 for consistency with the MARS scoring. The scores of the top 3 apps were compared with the mean score of all apps on each MARS item and subscale using two-tailed *t* tests.

The apps were then grouped based on app cost (free vs paid); the 6 paid apps averaged Can $3.66 (US $2.89) per app, with a range of Can $1.99-$6.99 (US $1.57-$5.52), and the group scores were compared among the MARS subscales. This analysis was repeated by grouping the apps into 2 categories reflecting the duration since their last update: apps last updated less than 6 months ago and apps updated more than 6 months ago.

Separate from the MARS scores, we conducted a quantitative examination of the types of apps available in each store. The apps were grouped into 3 functional categories, and these groups were compared among the 4 stores for significant differences using chi-square tests. The 3 app categories were informational, actionable, and consultatory. The apps were classified into 1 of these 3 groups based on their primary functionality. If an app contained elements of more than one category, we examined the app’s description to determine how the developers intended the app to be used. Informational apps primarily provided information about children’s illnesses, whereas actionable apps guided users to care recommendations, and consultatory apps provided a telehealth-based *virtual physician* service.

We also examined how the top 3 apps for childhood illnesses differed from the average app scores by dimension and scale item and compared them using *t* tests.

## Results

### Overview

**Figure 1:**
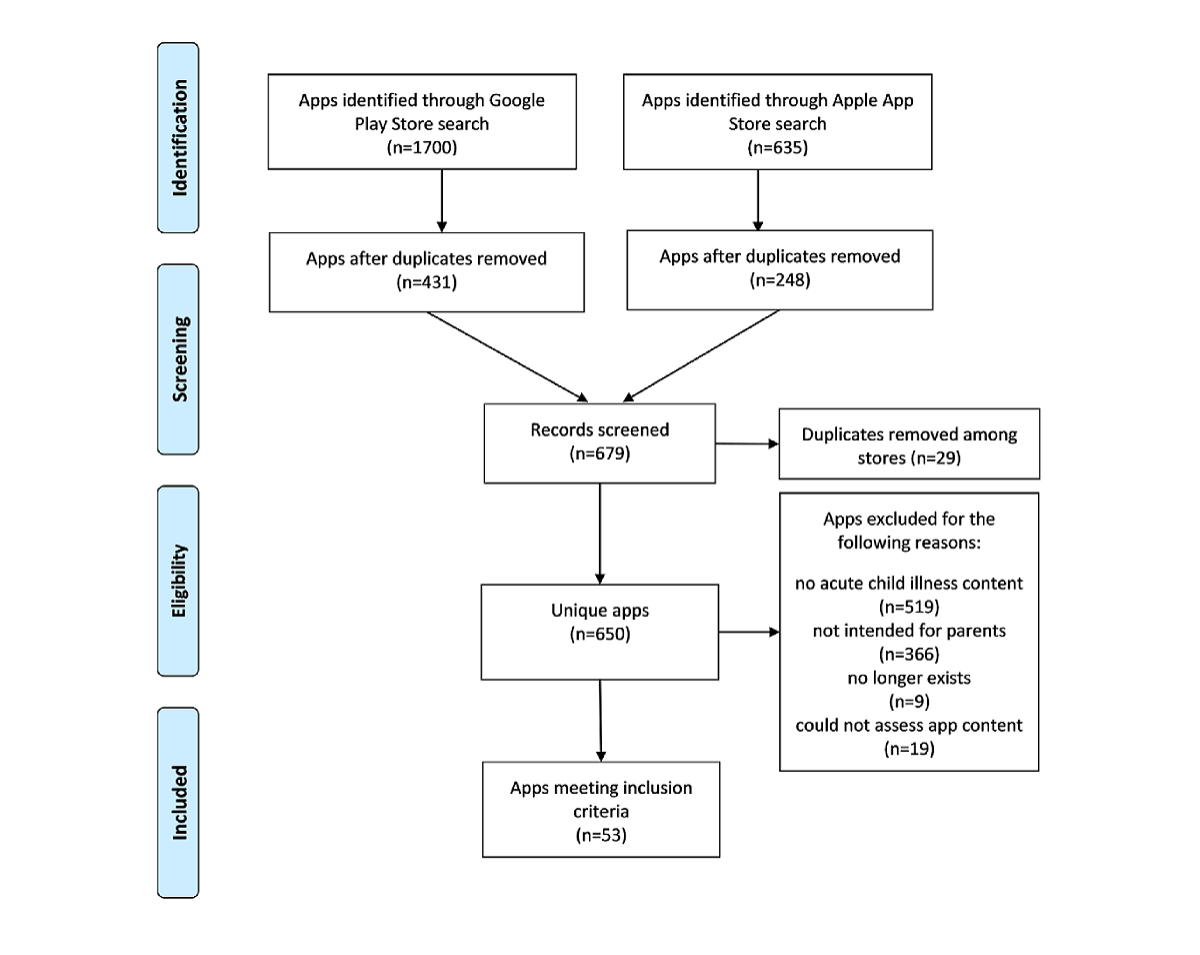
PRISMA (Preferred Reporting Items for Systematic Reviews and Meta-Analyses) diagram showing the environmental scan search and app screening process.

The PRISMA (Preferred Reporting Items for Systematic Reviews and Meta-Analyses) diagram (Figure 1) provides an overview of the search and screening process [50].

Our searches identified 2335 apps: 1700 (72.81%) from the Google Play Store and 635 (27.19%) from the Apple App Store. Of these 2335 apps, 650 (27.84%) were unique and 1685 (72.16%) were duplicates. After we applied our inclusion criteria to the 650 unique apps, 70 (10.8%) were considered potentially relevant; of these 70 apps, 17 (24%) were excluded, leaving 53 (76%) of apps for analysis. A total of 62% (33/53) of apps were available through the Google app marketplace and 55% (29/53) through the Apple app marketplace (9/53, 17%) of apps were available on both platforms).

### App Characteristics

All 53 apps focused on physical health: 19 targeted goal setting as a focus, whereas 18 targeted behavior change. A total of 50 apps had a theoretical background based in information or education delivery, 46 apps provided advice, tips, strategies, or skills training, 25 focused on assessment, and 17 dealt with information monitoring or tracking.

In all, 37 apps had a commercial affiliation, whereas 9 were government-affiliated, 3 had an affiliation with a nongovernmental organization, and one was affiliated with a university. Two apps—Asthma Action Hero and Pediatrics for All—did not fall into the MARS classification: the development of these apps was affiliated primarily with 2 individual physicians, Dr Helena K Bentley and Dr Hugo Rodrigues, respectively. Of the 53 apps assessed, 50 were targeted at young adults and adults, whereas one app was targeted at these groups as well as older adolescents, and 2 apps were targeted at general audiences, including children aged below 12 years. It should be noted that these findings are not in conflict with our inclusion criteria of an app’s target audience consisting of parents: apps can target multiple user ages while including parents as an intended audience, and all age groups included in the MARS (with the exception of children) could include parents.

In terms of app requirements and functionality, 8 apps allowed password protection, 23 had sharing features (eg, options for posting to Facebook or Twitter), 19 had a visible app community, 27 required internet access to load all parts of the app (including advertisements), and 7 required users to have an account and log in. A total of 9 apps did not seem to contain any of these features.

### MARS Ratings

The MARS subscales assess engagement using 5 items (mean 2.8/5, SD 0.86), functionality using 4 items (mean 4.0/5, SD 0.72), esthetics using 3 items (mean 3.1/5, SD 0.87), information quality using 7 items (mean 2.7/5, SD 1.32), and subjective quality using 4 items (mean 2.2/5, SD 0.79). The item scores for each dimension are summarized in Table 1.

**Table 1:** Mobile App Rating Scale item and subscale score averages for the assessed apps (N=53).

Subscale and item		Values, mean (SD)
**Engagement**		
	Entertainment	2.2 (0.57)
	Interest	3.1 (0.97)
	Customization	2.0 (1.07)
	Interactivity	3.3 (0.69)
	Target audience	3.7 (0.92)
	Engagement average	2.8 (0.86)
**Functionality**		
	Performance	4.0 (0.81)
	Ease of use	4.0 (0.68)
	Navigation	3.9 (0.52)
	Gestural design	4.1 (0.81)
	Functionality average	4.0 (0.72)
**Esthetics**		
	Layout	3.5 (0.75)
	Graphics	3.0 (1.08)
	Visual appeal	2.7 (0.72)
	Aesthetics average	3.1 (0.87)
**Information quality**		
	Description accuracy	4.3 (0.91)
	Goal setting	2.5 (2.24)
	Information quality	3.6 (0.95)
	Information quantity	3.0 (1.09)
	Visual information	3.0 (1.14)
	Credibility	2.6 (1.47)
	Evidence base	0.3 (0.89)
	Information average	2.7 (1.32)
**Subjective quality**		
	Recommended app	2.8 (1.31)
	Frequency of app use	1.5 (0.54)
	Willingness to pay for app	1.2 (0.71)
	Subjective star rating	3.3 (1.17)
	Subjective average	2.2 (0.79)

**Figure 2:**
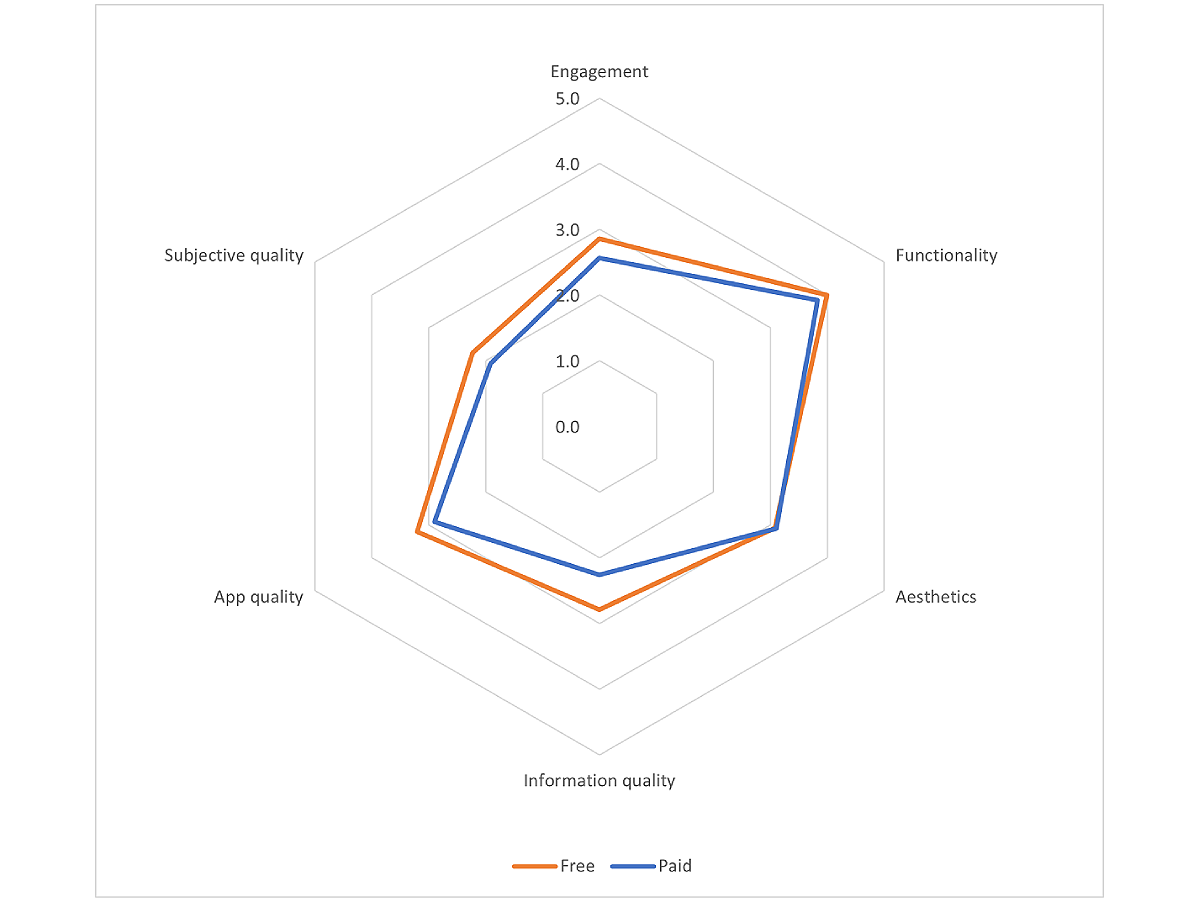
Comparison between paid and free app scores by Mobile App Rating Scale subscale.

Of the 53 apps assessed, the average user rating across all versions of all apps was 4.4/5, and the apps had been rated a total of 386,024 times (median 6, IQR 0-68). Because there was a large SD of 39,730 ratings, we normalized the rating by n ratings for a weighted average rating of 4.7/5 across all apps, excluding the apps without ratings. Of the 53 apps, 6 had to be purchased for use, and all other apps were free to use. The MARS subscale summary scores, grouped by paid versus free apps, are presented in Table 2. A comparison between the paid and free app scores by MARS subscale is shown in Figure 2. The free apps scored slightly higher on the overall MARS score and on 4 of the 5 subscales, although the differences were not significant.

**Table 2:** Mobile App Rating Scale subscale summary scores for app cost and duration since last update (N=53).

Mobile App Rating Scale subscale	Cost				Duration since update		
	Free (n=47), mean (SD)	Paid (n=6), mean (SD)	*P* value	Less than 6 months, mean (SD)		More than 6 months, mean (SD)	*P* value
Engagement	2.9 (0.63)	2.6 (0.61)	.32	3.0 (0.80)		2.7 (0.48)	.16
Functionality	4.0 (0.58)	3.8 (0.58)	.53	4.0 (0.41)		4.0 (0.49)	.95
Esthetics	3.1 (0.70)	3.1 (0.81)	.94	3.3 (0.51)		3.0 (0.35)	.24
Information quality	2.8 (0.80)	2.3 (0.74)	.15	2.9 (1.26)		2.6 (1.02)	.38
Subjective quality	2.2 (0.79)	1.9 (0.86)	.42	2.4 (0.75)		2.1 (0.54)	.32

The apps were last updated, on average, on June 19, 2019, with a range of most recent updates between February 9, 2016, and August 31, 2020. Of the 53 apps assessed, 20 (38%) had been updated within 6 months at the time the marketplace searches were conducted. The differences in the app scores when grouped by duration since the last update are shown in Table 2. Those updated within 6 months scored slightly higher overall and on 4 of the 5 MARS subscales, but the scores were not significantly different.

**Figure 3:**
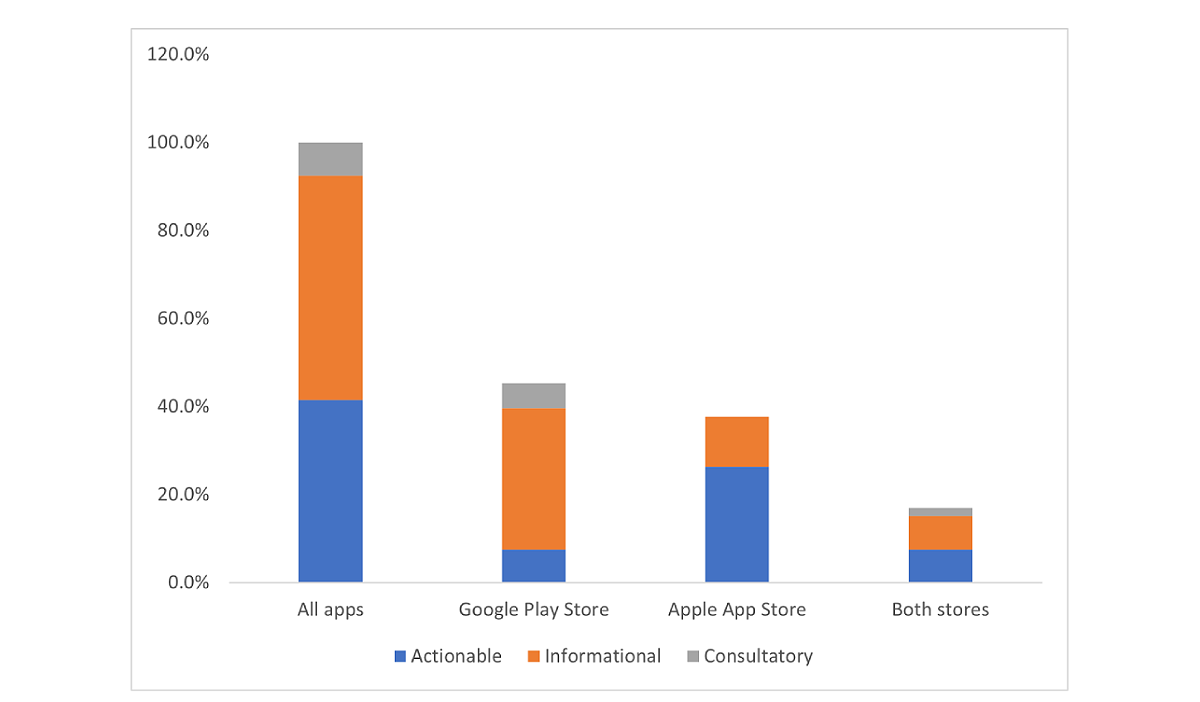
Types of apps available in each marketplace.

Outside of the MARS assessments, we found that all the assessed apps could be sorted into one of three categories: primarily informational (eg, e-books), actionable (eg, tools), and consultatory (eg, telemedicine). On examining the distribution of these 3 categories between the Apple and Google app marketplaces, we found that the 2 stores had a different division of app types, as shown in Table 3 and Figure 3.

**Table 3:** Proportion of acute childhood illness apps available by store, grouped by primary functional category (N=53).

		Actionable	Informational	Consultatory
**All apps, n (%)**		22 (42)	27 (51)	4 (8)
	Google Play Store	4 (8)	17 (32)	3 (6)
	Apple App Store	14 (26)	6 (11)	0 (0)
	Both app stores	4 (8)	4 (8)	1 (2)
Difference, *P* value		.002	.03	.08

**Figure 4:**
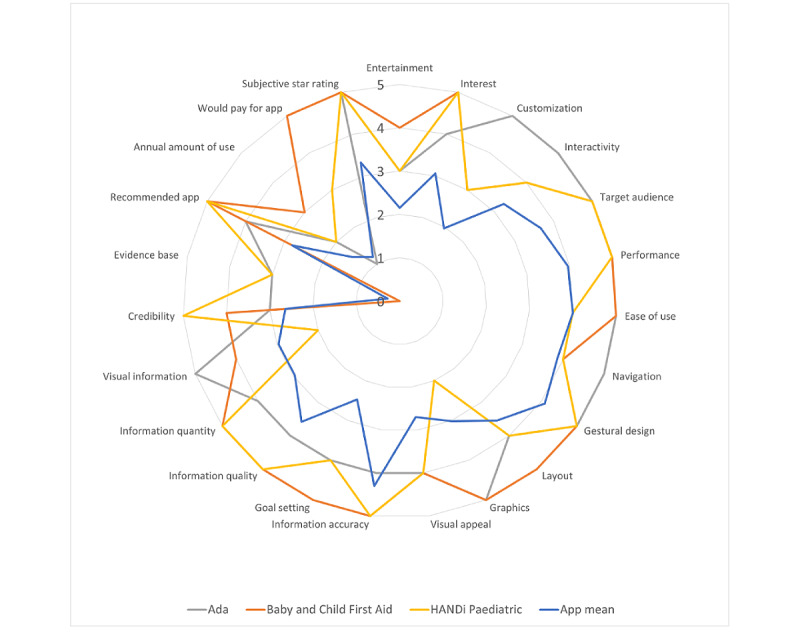
Mobile App Rating Scale app radar for top three apps versus the mean score of all 53 apps.

**Figure 5:**
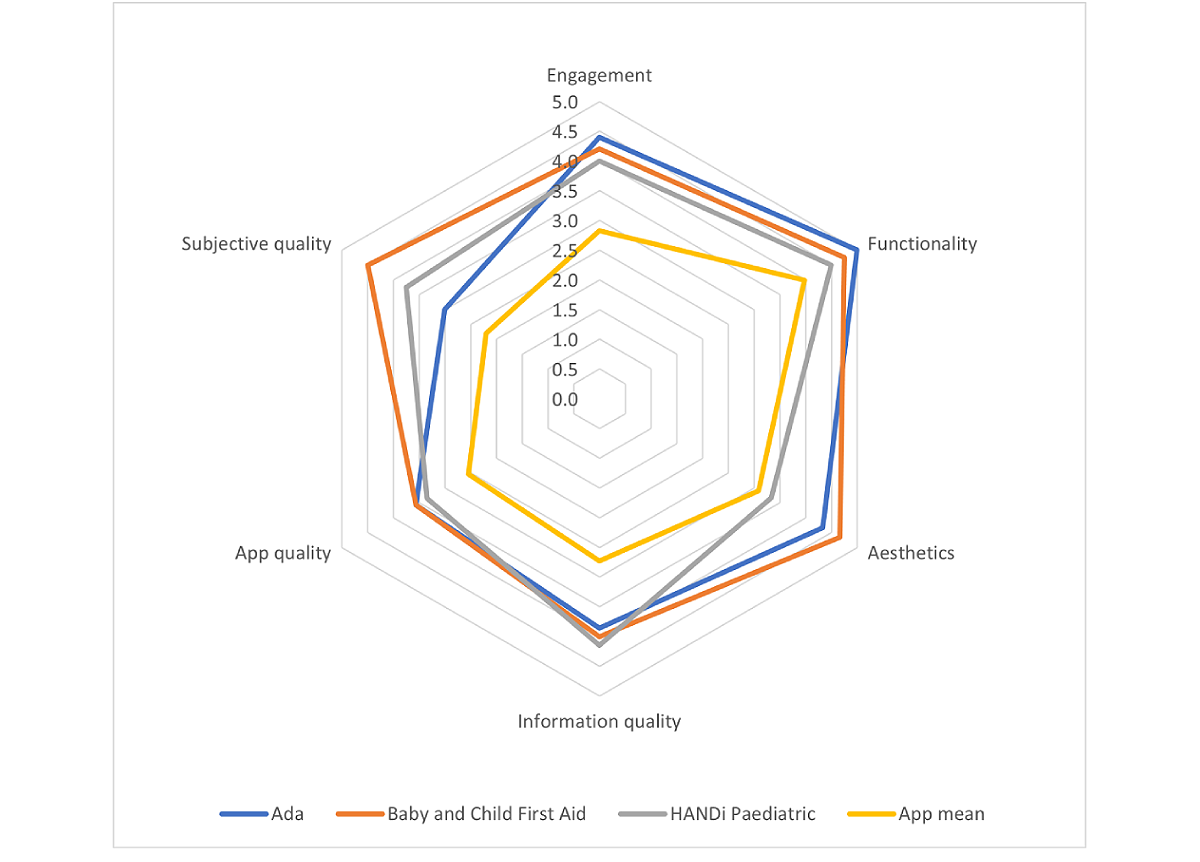
Radar graph of top apps by Mobile App Rating Scale subscale.

Of the 53 apps assessed, the top 3 on the MARS scale were Baby and Child First Aid by the British Red Cross (4.5/5), Ada by Ada Health (4.5/5), and HANDi Paediatric by Musgrove Park Hospital (4.2/5). Baby and Child First Aid provides common and emergency first aid scenarios and advice to parents. HANDi Paediatric has a similar focus on children’s health, but it provides illness-related information rather than first aid advice. Ada is an artificial intelligence–enabled symptom checker that asks a series of diagnostic questions to users to suggest illnesses, and it can be used for third parties (eg, children). A visual representation of all MARS items is presented in Figure 4, which shows the average rating of all apps (in blue), and a comparison of the 3 apps that had the top ratings on each subdimension of the MARS. The top 3 apps were also examined by MARS subscale scores (Table 4; Figure 5).

**Table 4:** Top apps by Mobile App Rating Scale subscale score.

Mobile App Rating Scale subscale	App mean (SD)	Subscale score				Top 3 mean versus app mean, *P* value
		Baby and Child First Aid	Ada	HANDi Paediatric		
Engagement	2.8 (0.86)	4.2	4.4	4	<.001	
Functionality	4 (0.72)	4.8	5	4.5	.01	
Esthetics	3.1 (0.87)	4.7	4.3	3.3	.11	
Information quality	2.7 (1.32)	4	3.9	4.1	<.001	
Subjective quality	2.2 (0.79)	4.5	3	3.8	.06	

Regarding the information provided within the apps, the source of information was often unlisted or unclear. However, we noted that some apps had matching information: 26% (14/53) of the apps contained content matching text from the Schmitt-Thompson Clinical Content nursing triage guidelines [51] and were similar in appearance and functionality. Predominantly, these apps were owned by individual children’s hospitals in the United States (10/14, 71%). The 4 other apps used guidelines developed by the UK-based National Health Service clinical commissioning groups; each was developed for region-specific deployment (eg, Suffolk).

### App Marketplaces

With regard to the differences between the app marketplaces, after pooling the results from both marketplaces, we noted that 4 apps were available in Canada but not in the United States, whereas 14 were available in the United States but not in Canada. However, with the exception of Baby and Child First Aid, the apps available in Canada but not in the United States were of low quality (mean MARS score 2.4/5, SD 0.20). Furthermore, of the apps available in the United States but not in Canada, 71% (10/14) were local hospital apps.

## Discussion

### Principal Findings

The purpose of conducting this environmental scan was to generate a comprehensive picture of the app landscape for parents in North America trying to make the best health decisions for their acutely ill child and for health care researchers to identify gaps in the app ecosystem. Given the observed trend of parents not seeking health care services for their children during the COVID-19 pandemic [7], it is important to address the gap we observed in Canadian context–specific evidence-based information to support parental health care decisions for their children. With the large changes in pediatric ED use during the COVID-19 pandemic [7], there is a need to create information access that identifies when interventions are necessary, rather than the current trend of parents delaying treatment of their children’s less serious illnesses.

Our scan of the Apple App Store and Google Play Store in Canada and the United States identified 53 apps that we appraised using the validated MARS tool. The mean MARS score was 3.2/5 (SD 0.60), scoring slightly higher than the scale’s *average* category of 3/5. The apps had a high overall functionality score, with especially strong gestural design (ie, whether interactions such as taps and other screen-based gestures in the app were consistent and intuitive when examining all app pages). Gestures worked in the apps with few errors. The esthetics score rated slightly below the app mean score, primarily because of a low visual appeal score, but it was bolstered by strong app layouts.

The app design choices were often outdated, an unsurprising finding considering the long average update time of 453 days. If only those apps that were updated within 6 months are considered, the visual appeal score of the 20 included apps is slightly higher (although not significantly so). The low average information quality of apps was caused primarily by a low evidence base score (ie, proof that the app has been trialed, as verified by evidence in published scientific studies). This seems to diverge from mHealth app studies in other health disciplines such as pharmacy, which found that information quality had the highest scores among the subscales [52]. When we examined the literature for occurrences of app names, we found mention of only 4 apps: Ada, Kinsa (a digital thermometer companion), HANDi Paediatric, and WebMD Baby. Information quality was buoyed by a strong description accuracy (mean 4.3, SD 0.91; ie, whether the app’s described content matched its actual content) and information quality (mean 3.6, SD 0.95). The average scores in subjective quality app ratings (mean 3.3, SD 1.16) were similar to the overall assessed scores, whereas willingness to pay for an app (mean 1.2, SD 0.71) and projected app use (mean 1.5, SD 0.54) were low. However, these low scores may better reflect the fact that most of the apps were designed primarily for ad hoc use during acute illnesses, rather than for regular use in chronic health situations.

We found that the apps included in each store seemed to have different foci. On closer examination, the Google marketplace apps were noted to be primarily information-focused (eg, e-books), with 4 times as many (17/53, 32%) information-focused apps as action-oriented apps (eg, symptom-tracking tools; 4/53, 8%). The Apple marketplace apps had the opposite trend: there were more than twice as many action-oriented apps (14/53, 26%) as information-focused apps (6/53, 11%). This finding suggests that a parent’s choice of mobile device brand may not be incidental: beyond this choice leading to different information availability, there is a difference in the format of information presented. Specifically, unless parents have access to both Android and Apple devices, they will only have access to apps available on one app store. Depending on the device available, these differences may affect how effectively parents are able to find and use an app to help their child. For example, the Apple and Google app stores differ in how apps are assessed for quality and how their search algorithms work, as well as in quality requirements for app developers [53]. As has been previously suggested, the mode in which information is presented matters [54-56], and this difference has the potential to affect how parents learn more about their child’s illness. In the same manner, the fact that there are apps that are available in one country but not in another also affects parents’ ability to find health information. User characteristics also present a possible barrier to accessing health information: a Dutch study concluded that app use differed based on user demographics (eg, app use was higher in younger, more educated users) [57].

When we examined app pay practices, we found that the paid apps scored slightly lower (although not significantly so, based on two-tailed *t* tests assuming equal variances based on similar domain and overall variances) on 4 of the 5 MARS subscales—engagement (*P*=.29), functionality (*P*=.51), information quality (*P*=.13), and subjective quality (*P*=.36)—and higher on one subscale: aesthetics (*P*=.93). Limiting the interpretation of this finding is the small number of paid apps compared with that of free apps (6 vs 47, respectively). We also examined whether the apps that were updated more than 6 months ago differed in quality from the more recently updated apps. Although only small differences were seen among the apps, it will be interesting to investigate in future studies whether, as noted with previous work [58], some apps worsen with time in domains such as privacy. Trust is an increasingly important dimension of apps, given their status as an emerging health technology [59], and it has been shown to be an important consideration in app adoption in some groups such as the Deaf [60]. A limitation of the MARS is that it does not assess app credibility to the depth suggested by other app assessment frameworks such as the mHealth App Trustworthiness checklist [59]. Future research could assess apps using such a checklist to discover how items important to accessibility, such as customization, correlate with app trustworthiness dimensions such as user autonomy and empowerment.

On examining the top 3 apps, we noted that Baby and Child First Aid presents 17 common first aid scenarios (eg, *allergic reaction*) to parents, with a set of causes and steps to follow (eg, basic triage, ideal action to take, and action to take in an emergency). This app also allows parents to self-assess the effectiveness of the teaching videos and other materials available through quizzes. This app is highly relevant, comes from a credible organization, and is updated regularly (at the time of writing, the most recent update was within 6 months, on October 12, 2020). However, this app’s credibility could be improved by demonstrating its effectiveness in peer-reviewed studies. Ada is an artificial intelligence–based symptom checker that uses a series of questions to reach a list of possible diagnoses, ranked in order of likelihood. It provides options to check another person’s symptoms, track symptoms, and centralize health information (eg, medications and insurance). Ada benefits from a presence in scientific studies [61,62]. HANDi Paediatric provides parents with home assessment guidelines and information about common childhood illnesses. Notably, it includes nearby ED locations and the ability to call emergency services if needed. Taken together, these 3 apps give parents information related to emergency pediatric first aid, identification of (and appropriate response to) common childhood illnesses, 2 ways to check symptoms, and a means of responding to emergency situations. Two of the top apps (Ada and HANDi Paediatric) have a substantial, published evidence base [63-65], and there is a significant difference between their scores and the mean score of all apps for this item (Ada=3/5, HANDi Paediatric=3/5; item mean 0.28/5, SD 0.89; *P*<.001). A comparison of these top 3 apps with the app average shows that the top 3 apps are significantly different in terms of engagement (*P*<.001), information quality (*P*<.001), and functionality (*P*=.01), with no significant differences for subjective quality and esthetics.

Among all the apps reviewed, there was a lack of app presence in published studies (MARS item evidence base: Has the app been trialed or tested? Must be verified by evidence [in published scientific literature]; mean 0.28/5, SD 0.89). However, information quality and quantity were above average and average (mean scores 3.6 and 3.0, respectively). Therefore, there seems to be a gap between the app’s provision of quality content and the ability to make published claims about the effectiveness of accessing that content. This is an area that has been examined previously: a 2014 study found that there was no crossover between academic and commercial app offerings for pain-related apps [35]. The lack of rigor in assessing apps gave rise to the concern that a lack of peer-reviewed apps has resulted in an unregulated and uncertified set of apps available to consumers and that this could be addressed by scientists leveraging decreasing app development times and costs to offer more evidence-supported apps [66]. As one recent investigation into symptom checkers concluded, apps must be assessed on the accuracy of their results in the context of coverage of available conditions and patient populations if these apps do not give results for some subgroups (eg, children) [67].

In assessing these apps, a key gap for parents to be aware of, and for developers to note, was the lack of Canadian-developed content available to parents. Although apps for children’s illnesses were developed using knowledge from US and UK sources, there were no apps that seemed to use Canadian-based information. Among the top 3 apps, Ada develops medical content through an internally peer-reviewed process, using a team of physicians in the United States [68]. HANDi Paediatric bases its information on consultants and general practitioner and hospital clinical guidelines in the United Kingdom [69]. Baby and Child First Aid does not list a source of information. This strongly suggests that although high-quality information for childcare is available in apps, Canadian parents must rely on guidelines developed elsewhere. It also means that Canadian interests, values, and approaches to pediatric illnesses are not well represented in the app marketplaces. This lack of representation is concerning and suggests a gap in availability of an app for parents based on Canadian evidence-based health guidelines.

### Limitations

When we scrutinized the app stores, we found that the Google Play Store does not recommend similar apps if the app being examined is not compatible with the user’s devices, and the Apple App Store does not show apps that are unavailable on the user’s current device. Spellings and idioms were important: searching *pediatric* returns apps from North America first, whereas *paediatric* returns UK- and Australia-based apps. The mechanisms behind each store’s search parameters are unique and undisclosed, and the differences in search mechanisms that are described do not support the use of a single systematic process used to search both stores. Therefore, there is no way to guarantee that all relevant and currently available apps have been captured during the Play Store and App Store searches. We attempted to mitigate this limitation through our use of multiple search terms co-designed with a research librarian and having 2 reviewers assess all apps for inclusion. In addition, the testing of each app was conducted by 1 evaluator (with a second evaluator assessing 5/53, 10% of apps); having more evaluators comparing ratings would decrease the possibility that the rating agreements occurred by chance. Our examination of peer-reviewed study availability relating to single apps was limited to a single search engine (Google Scholar), and rigor could have been added to this method by triangulating results with PubMed and directly checking developer websites.

### Conclusions

This study examined 53 apps for parents related to acute childhood illness using the MARS assessment tool. Overall app quality was rated slightly above average, driven by high scores in functionality, whereas there was scope for the apps to improve their information content through increased presence in peer-reviewed studies. There was a strong need identified for evidence-based apps of Canadian origin. These apps should be developed in a way that ensures accessibility of content, transparency of information sources, and effectiveness as demonstrated through peer-reviewed studies. In the case that developers are unable to test for effectiveness, demonstrating that the app was created using evidence-based, peer-reviewed information would give parents a basis for trusting the app’s content. Considering these findings in relation to the current practice of most parents of using internet searches as their clinician-adjunct source of health information, there is a clear opportunity for clinicians and scientists to collaborate to create apps that increase parents’ confidence in their health knowledge.

## Appendix

Multimedia Appendix 1 App quality ratings by Mobile App Rating Scale domain.

## Abbreviations

ED: emergency department
MARS: Mobile App Rating Scale
mHealth: mobile health
PRISMA: Preferred Reporting Items for Systematic Reviews and Meta-Analyses
