# Supplementary material for: Characteristics of Acute Childhood Illness Apps for Parents: Environmental Scan
Source: J Med Internet Res. 2021 Oct 19;23(10):e29441. doi: 10.2196/29441 (PMC8564653; doi:10.2196/29441)
Supplement: Multimedia Appendix 1 [file jmir_v23i10e29441_app1.doc]

## Appendix

Table S1. MARS App Characteristics

| App Name | N ratings all versions | Affiliations |  |
| --- | --- | --- | --- |
| Rating this version | Version | Age group |  |
| Rating all versions | Last update | Platform |  |
| Developer | Focus: what the app targets | Brief description |  |
| N ratings this version | Theoretical background/Strategies | Cost |  |

Table S2. MARS App Quality Questions by Subscale

| **Subscale** | **Item** | **Questions** |
| --- | --- | --- |
| Engagement | Entertainment | Is the app fun/entertaining to use? |
|  | Interest | Is the app interesting to use? |
|  | Customisation | Does it provide/retain all necessary settings/preferences? |
|  | Interactivity | Does it allow user input, provide feedback, contain prompts? |
|  | Target group | Is the app content appropriate for your target audience? |
| Functionality | Performance | How quickly do the app features work? |
|  | Ease of use | How easy is it to learn how to use the app? |
|  | Navigation | Is moving between screens logical/appropriate? |
|  | Gestural design | Are interactions consistent and intuitive? |
| Aesthetics | Layout | Is arrangement and size of content on the screen appropriate? |
|  | Graphics | How high is the quality/resolution of graphics used? |
|  | Visual appeal | How good does the app look? |
| Information | Accuracy of description | Does app contain what is described? |
|  | Goals | Does app have specific, measurable and achievable goals? |
|  | Quality of information | Is app content correct, well written, and relevant? |
|  | Quantity of information | Is the extent coverage within the scope of the app; and comprehensive but concise? |
|  | Visual information | Is visual explanation of concepts clear, logical, correct? |
|  | Credibility | Does the app come from a legitimate source? |
|  | Evidence base | Has the app been trialled/tested? |
| Subjective Quality | Recommended | Would you recommend this app to people who might benefit from it? |
|  | Usage | How many times do you think you would use this app in the next 12 months if it was relevant to you? |
|  | Cost | Would you pay for this app? |
|  | Stars | What is your overall star rating of the app? |

Table S3. App Quality Ratings by MARS Domain

|  | **Engagement** | **Functionality** | **Aesthetics** | **Information** | **Score** |
| --- | --- | --- | --- | --- | --- |
| **App Name** |  |  |  |  |  |
| Ada - your health companion | 4.4 | 5.0 | 4.3 | 3.9 | 4.5 |
| Akron Children's Anywhere | 3.4 | 4.0 | 3.7 | 3.1 | 3.6 |
| All Pediatric Diseases | 2.2 | 4.0 | 3.0 | 2.3 | 2.8 |
| Arkansas Children's | 3.4 | 4.0 | 3.7 | 3.1 | 3.6 |
|  |  |  |  |  |  |
| Asthma Action Hero | 3.4 | 4.3 | 3.3 | 3.3 | 3.6 |
| Baby and Child First Aid | 4.2 | 4.8 | 4.7 | 4.0 | 4.5 |
| Baby Illness, Prevention, Cure | 1.8 | 4.8 | 2.3 | 1.4 | 2.4 |
| Babygogo Parenting - Baby Care & Pregnancy Tips | 3.0 | 3.8 | 3.3 | 1.7 | 2.8 |
| Berkshire Child Health | 2.6 | 4.8 | 3.0 | 3.1 | 3.4 |
| Cheshire Child Health | 2.6 | 4.8 | 3.0 | 3.1 | 3.4 |
| Child Health Guide Newham | 2.6 | 4.8 | 3.0 | 3.1 | 3.4 |
| Child Health HMR | 2.6 | 4.8 | 3.0 | 3.1 | 3.4 |
| Child Symptom Checker | 2.6 | 4.0 | 3.7 | 2.3 | 3.0 |
| ChildHealthGuideWF | 2.6 | 4.8 | 3.0 | 3.1 | 3.4 |
| Children/Pediatric/Emergency/Parenting /Nursing | 1.6 | 3.0 | 2.0 | 1.7 | 2.1 |
| Children's Hospital | 2.2 | 2.5 | 2.7 | 3.1 | 2.8 |
| Children's On Call | 3.4 | 4.0 | 3.7 | 3.1 | 3.6 |
| ChildrensLA | 3.2 | 3.8 | 3.7 | 3.3 | 3.5 |
| ChildrensMD | 3.4 | 4.0 | 3.7 | 3.1 | 3.6 |
| ChildrensPgh | 3.4 | 4.0 | 3.7 | 3.1 | 3.6 |
| Doctor At Home | 2.0 | 3.8 | 2.3 | 1.6 | 2.3 |
| Doctor At Home: Home Treatment | 2.2 | 3.5 | 2.3 | 1.7 | 2.4 |
| Guide to Newborn Care | 2.4 | 3.8 | 2.0 | 1.9 | 2.5 |
| HANDi Paediatric | 4.0 | 4.5 | 3.3 | 4.1 | 4.2 |
| Help for Kids Health and Diet | 2.4 | 4.3 | 1.7 | 1.9 | 2.6 |
| Home Remedies, Natural Cures & Herbal Treatment | 2.6 | 4.0 | 2.0 | 1.0 | 2.3 |
| Home Remedies+ : Natural Cures | 2.0 | 4.0 | 2.0 | 1.6 | 2.3 |
| Kid Care-St. Louis Children's | 3.4 | 4.0 | 3.7 | 3.1 | 3.6 |
| Kids Health Info | 3.0 | 3.0 | 3.0 | 3.1 | 3.2 |
| KidsDoc - from the AAP | 3.2 | 4.0 | 3.7 | 3.1 | 3.5 |
| Kinsa for Wireless Smart Thermometers | 3.2 | 4.3 | 4.3 | 3.9 | 4.0 |
| MD 4KIDS | 3.4 | 4.0 | 3.7 | 3.1 | 3.6 |
| Moab rehydration app | 2.4 | 4.0 | 2.3 | 2.1 | 2.7 |
| Newborn Baby Care & Advices | 2.0 | 3.8 | 1.7 | 1.6 | 2.2 |
| NHS Sunderland Common Childhood Illnesses | 2.6 | 4.0 | 3.0 | 2.0 | 2.8 |
| OK Kids MD | 3.4 | 4.0 | 3.7 | 3.1 | 3.6 |
| Oldham Child Illness | 2.6 | 4.8 | 3.0 | 3.1 | 3.4 |
| Parenting - Pediatric Oncall | 2.4 | 2.8 | 2.0 | 2.3 | 2.4 |
| PediaCHeck | 3.4 | 4.0 | 3.7 | 3.1 | 3.6 |
| Pediatric Disease and Treatment | 2.4 | 3.3 | 2.7 | 2.4 | 2.7 |
| Pediatric Disease and Treatment (Free) | 2.0 | 4.0 | 2.0 | 1.6 | 2.3 |
| Pediatric SymptomMD | 3.4 | 4.0 | 3.7 | 3.1 | 3.6 |
| Pediatrics for all | 2.0 | 4.5 | 3.3 | 1.4 | 2.6 |
| PedsPartner | 3.4 | 4.0 | 3.7 | 3.1 | 3.6 |
| Pregnancy & Baby Tracker | 2.4 | 3.0 | 3.0 | 2.0 | 2.6 |
| RD - Childhood Skin Rashes | 2.4 | 4.0 | 2.7 | 2.3 | 2.8 |
| Stanford Children's Health | 2.6 | 3.0 | 3.3 | 3.3 | 3.2 |
| Suffolk Child Health | 2.6 | 4.8 | 3.0 | 3.1 | 3.4 |
| SymptomMD | 3.4 | 4.0 | 3.7 | 3.1 | 3.6 |
| Symptoms Checker | 2.2 | 2.8 | 2.7 | 2.0 | 2.4 |
| The Lullaby Trust Baby Check | 2.8 | 3.8 | 3.3 | 4.0 | 3.6 |
| WebMD Baby | 3.4 | 4.3 | 3.3 | 4.0 | 3.9 |
| Wesley | 3.4 | 4.0 | 3.7 | 3.1 | 3.6 |

Table S4. App Descriptions

|  | **App Name** | **Description** |
| --- | --- | --- |
| 1 | Ada - your health companion | AI-enabled symptom checker |
| 2 | Akron Children's Anywhere | Child health management (including symptom checker, first aid reference, hospital locations, medication dosing) |
| 3 | All Pediatric Diseases | Infant and child viral infection information |
| 4 | Arkansas Children's | Child health management (including symptom checker, first aid reference, hospital locations, medication dosing) |
| 5 | Asthma Action Hero | Notification-based asthma management |
| 6 | Baby and Child First Aid | Child health information and learning tools |
| 7 | Baby Illness, Prevention, Cure | Identification and treatment of common infant illnesses |
| 8 | Babygogo Parenting - Baby Care & Pregnancy Tips | Pregnancy, infancy, and child development info and tools |
| 9 | Berkshire Child Health | Baby and child common illness management |
| 10 | Cheshire Child Health | Baby and child common illness management |
| 11 | Child Health Guide Newham | Baby and child common illness management |
| 12 | Child Health HMR | Baby and child common illness management |
| 13 | Child Symptom Checker | Symptom-based child health search linked to Wikipedia articles |
| 14 | ChildHealthGuideWF | Baby and child common illness management |
| 15 | Children/Pediatric/Emergency/Parenting /Nursing | Parent/nurse/clinician tools and information database of childhood illnesses |
| 16 | Children's Hospital | Hospital-specific app with children’s health info and access to emergency numbers/hospital locations |
| 17 | Children's On Call | Child health management (including symptom checker, first aid reference, hospital locations, medication dosing) |
| 18 | ChildrensLA | Hospital-specific platform focused on communication with care team, access to non-sensitive records, symptom checker |
| 19 | ChildrensMD | Child health management (including symptom checker, first aid reference, hospital locations, medication dosing) |
| 20 | ChildrensPgh | Child health management (including symptom checker, first aid reference, hospital locations, medication dosing) |
| 21 | Doctor At Home | Natural remedies (e.g. applying parsley to a bruise) for home treatment of diseases. |
| 22 | Doctor At Home: Home Treatment | Natural remedies (e.g. applying parsley to a bruise) for home treatment of diseases. |
| 23 | Guide to Newborn Care | Infant care guides, common infant medical conditions, information on when to call a doctor |
| 24 | HANDi Paediatric | Expert-developed app and assessment guidelines for common childhood illnesses |
| 25 | Help for Kids Health and Diet | Diet-focused app with information and remedies about common children’s illnesses |
| 26 | Home Remedies, Natural Cures & Herbal Treatment | Herbal treatment of illnesses, claims effectiveness at improving children’s immunity |
| 27 | Home Remedies+ : Natural Cures | Home remedies for illnesses with ability to ask questions on the app and submit remedies |
| 28 | Kid Care-St. Louis Children's | Child health management (including symptom checker, first aid reference, hospital locations, medication dosing) |
| 29 | Kids Health Info | Fact sheets on children and adolescent health, and hospital-specific services |
| 30 | KidsDoc - from the AAP | AAP Parenting website-linked app providing symptom-based medical advice, and information |
| 31 | Kinsa for Wireless Smart Thermometers | Bluetooth thermometer-linked app providing age- and temperature-specific fever guidance |
| 32 | MD 4KIDS | Care guides for children’s illness and injury designed to assist parents making care decisions |
| 33 | moab rehydration app | Age-specific oral rehydration guidance for children experiencing dehydration |
| 34 | Newborn Baby Care & Advices | General guides on newborn infant care, including illnesses and information on when to contact a pediatrician |
| 35 | NHS Sunderland Common Childhood Illnesses | Sunderland-specific app with information for managing childhood illness and locations to access health care services |
| 36 | OK Kids MD | Care guides for children’s illnesses, common questions asked by parents, and symptom checker |
| 37 | Oldham Child Illness | Baby and child common illness management |
| 38 | Parenting - Pediatric Oncall | Childhood disease information, alternative medicine, tools for parents, and vaccination information |
| 39 | PediaCHeck | Child and adolescent health management (including symptom checker, hospital locations, parent advice FAQs) |
| 40 | Pediatric Disease and Treatment | E-book intended for mothers and health care professionals, with quizzes on pediatric nursing |
| 41 | Pediatric Disease and Treatment (Free) | Information on childhood diseases to educate parents and help inform health care decisions |
| 42 | Pediatric SymptomMD | Child health management (including symptom checker, first aid reference, hospital locations, medication dosing) |
| 43 | Pediatrics for all | Information on common pediatric symptoms of illness, diseases, medications, and dose converter |
| 44 | PedsPartner | Children’s hospital-specific app (including symptom checker, first aid reference, hospital locations, medication dosing) |
| 45 | Pregnancy & Baby Tracker | Baby growth and development tracking, articles/videos on children’s health and illness, community of parents |
| 46 | RD - Childhood Skin Rashes | Diagnosis, description, alternate conditions, and treatment of childhood skin rashes |
| 47 | Stanford Children's Health | Hospital-specific app focused on patient engagement and education on children’s health conditions |
| 48 | Suffolk Child Health | Baby and child common illness management app focused on prevention of childhood illness |
| 49 | SymptomMD | Family health management (including symptom checker, first aid reference, Zocdoc (doctor finder) integration) |
| 50 | Symptoms Checker | Common illnesses app linking symptoms to etiology |
| 51 | The Lullaby Trust Baby Check | Set of health checks to test for signs of illness in infants and decide on level of care needed based on symptom severity |
| 52 | WebMD Baby | Baby development tracking, physician-reviewed advice on illness/emergencies, preparation for pediatrician visits |
| 53 | Wesley | Hospital-specific app with symptom checker, symptom decision chart, and self-care advice |
